# Iron-Sulfur Clusters: A Core Hub in Insect Energy, Reproduction, and Molting

**DOI:** 10.3390/insects17080791

**Published:** 2026-07-29

**Authors:** Xiao-Han Di, Shuo Liu, Duo Wang, Jing-Ze Liu

**Affiliations:** Ministry of Education Key Laboratory of Molecular and Cellular Biology, Hebei Key Laboratory of Animal Physiology, Biochemistry and Molecular Biology, Hebei Collaborative Innovation Center for Eco-Environment, Hebei Research Center of the Basic Discipline of Cellular Biology, College of Life Sciences, Hebei Normal University, Shijiazhuang 050024, China; dixiaohan0523@163.com (X.-H.D.); 17736501126@163.com (S.L.)

**Keywords:** iron-sulfur clusters, insect physiology, energy metabolism, reproduction, molting, magnetoreception

## Abstract

Iron-sulfur clusters are a class of functionally diverse protein cofactors. In recent years, significant progress has been made in insect research on iron-sulfur clusters, revealing that they serve as a core molecular hub that integrate multiple physiological processes. This review systematically summarizes the structural characteristics and biosynthetic pathways of iron-sulfur clusters, with an emphasis on insect-specific features in the stability of iron-sulfur cluster assembly proteins, cluster transfer mechanisms, and functional differentiation of ferredoxins. On this basis, it further elucidates the central roles of insect iron-sulfur clusters in energy homeostasis, reproductive capacity, ecdysis, detoxification metabolism, and magnetoreception, and revealed the potential regulatory mechanisms of iron–sulfur proteins in the aforementioned physiological processes in insects, including the electron transport chain, the tricarboxylic acid cycle, the AMPK/TOR signaling axis, cell cycle regulation, and P450 enzyme activity. These findings provide important theoretical foundations and potential molecular targets for the development of environmentally friendly pest control strategies with low resistance risk.

## Simple Summary

Iron-sulfur clusters are key protein cofactors in insects, participating in multiple physiological processes including energy production, reproduction, molting, and magnetic sensing. This review summarizes the structural features and biosynthetic pathways of iron-sulfur clusters, with a focus on the specific mechanisms underlying the stability of iron-sulfur cluster assembly proteins in insects. It also discusses the potential regulatory mechanisms by which iron-sulfur proteins function through pathways such as the electron transport chain, the TCA cycle, the AMPK/TOR signaling axis, cell cycle regulation, and cytochrome P450 enzyme activity.

## 1. Introduction

Iron-sulfur clusters (Fe-S clusters) are a class of highly conserved and widely distributed protein cofactors. Since the discovery of the first Fe-S protein, bacterial ferredoxin, in 1962, research on Fe-S clusters has expanded across diverse fields of eukaryotic biology [1,2]. Fe-S clusters, composed of a relatively multiple arrangement of iron ions and inorganic sulfur in specific three-dimensional configurations, demonstrate remarkable functional diversity [3]. Fe-S clusters, as protein cofactors, perform essential roles through four primary mechanisms: electron transfer, catalytic center formation, structural support, and environmental sensing. These functions underpin critical biological processes, including protein translation, DNA replication and repair, gene expression regulation, and oxidative stress responses [4,5,6].

The insect class is noteworthy for its remarkable biodiversity, encompassing a diverse array of species [7]. In recent years, significant progress has been made in the study of the biosynthesis pathways and physiological functions of Fe-S proteins in insects [8]. This progress can be attributed to the widespread application of functional RNA interference (RNAi) and omics technologies [9,10]. A mounting body of evidence suggests that Fe-S proteins may have a regulatory function that has hitherto been underestimated and that is critical to the specific life processes of insects. Research findings indicate that the disruption of the expression of genes involved in Fe-S clusters assembly can trigger a series of pleiotropic phenotypes in insects, including energy metabolism imbalance, reduced reproductive capacity, molting disorders, and impaired magnetic sensing ability. This multifaceted physiological disruption strongly suggests that Fe-S clusters may function as key protein cofactors, integrating and coordinating multiple vital activities in insects. Consequently, Fe-S clusters and their biosynthetic pathways and Fe-S proteins are increasingly recognized as highly promising novel molecular targets in pest control. Despite these advances, the precise molecular mechanisms by which Fe-S proteins regulate insect physiology remain to be fully elucidated, particularly in relation to their roles in complex processes such as reproduction and ecdysis. Concurrently, extant review literature primarily focuses on mammalian and yeast models [11,12,13,14], with a paucity of systematic summaries for the insect class. This review aims to elucidate how iron-sulfur proteins coordinate the diverse physiological functions in insects, emphasize their central role as integrative hubs in insect biology, and identify potential targets and research directions for the development of novel broad-spectrum pest control strategies with low resistance risk.

## 2. Structure of Iron-Sulfur Clusters in Proteins

**Figure 1:**
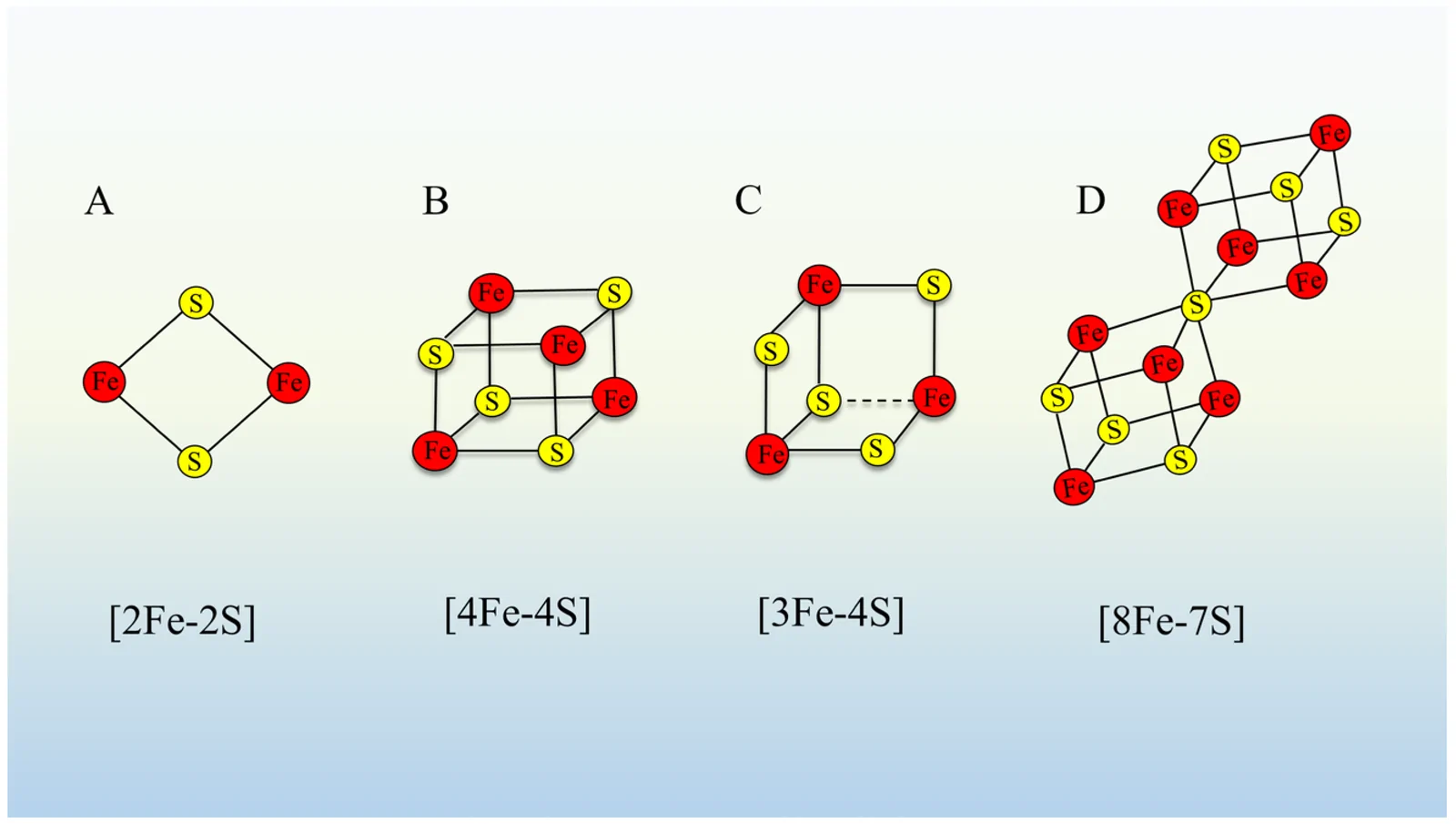
Schematic diagram of common structures of Fe-S clusters. (**A**) [2Fe-2S], (**B**) [4Fe-4S], (**C**) [3Fe-4S], (**D**) [8Fe-7S].

Fe-S clusters are metal clusters formed by iron atoms coordinated with inorganic sulfur atoms through coordination bonds. In living organisms, they perform various critical physiological functions such as electron transfer, catalytic reactions, and signal transduction [15]. To accommodate their functional diversity, Fe-S clusters exhibit significant structural variation. The [2Fe-2S] cluster consists of two iron atoms and two sulfur atoms arranged in a rhombic structure, with the two iron atoms typically coordinated by the thiol groups of four cysteine residues in total (i.e., each iron is coordinated by two cysteine residues) (Figure 1A). In Rieske-type structures, one iron atom is coordinated by two histidine residues, while the other iron atom is coordinated by two cysteine residues. The [2Fe-2S] cluster is primarily found in ferredoxins and Rieske iron-sulfur proteins, where it participates in electron transfer processes [16,17,18,19]. The [4Fe-4S] cluster can be viewed as a structural combination of two [2Fe-2S] clusters, which is the most widely distributed and functionally diverse type in iron-sulfur proteins. The [4Fe-4S] cluster possesses a cubane-like structure, with four iron atoms and four sulfur atoms arranged alternately to form a symmetric tetrahedral coordination environment, making it an ideal “electron carrier” (Figure 1B). It is widely distributed in respiratory chain complexes, nitrogenases, and photosystem I, playing key roles in catalysis, sulfur transfer, and signal sensing [20,21,22,23]. Building upon the [4Fe-4S] cluster, nature has also evolved other structural variants. The [3Fe-4S] cluster can be considered an analog of the [4Fe-4S] cluster with one iron atom missing (Figure 1C). Its asymmetric coordination environment and lower stability limit its distribution in nature [24,25]. The more complex [8Fe-7S] cluster, also known as the P-cluster, can be described as two [4Fe-4S] subclusters bridged by a shared sulfur atom (Figure 1D). It has so far been found exclusively in the MoFe protein of nitrogenases, where it efficiently transfers electrons, significantly enhancing nitrogen fixation efficiency [26,27]. Additionally, some microbial systems contain hybrid Fe-S clusters incorporating heteroatoms such as nickel and molybdenum, which catalyze important chemical reactions like carbon monoxide oxidation and nitrogen reduction [28,29,30]. Given the structural fundamentality of the [2Fe-2S] cluster and the functional diversity and high conservation of the [4Fe-4S] cluster, their corresponding assembly proteins and biosynthesis pathways have become research hotspots in the field of Fe-S clusters in recent years.

## 3. Biosynthesis of Iron-Sulfur Clusters

**Figure 2:**
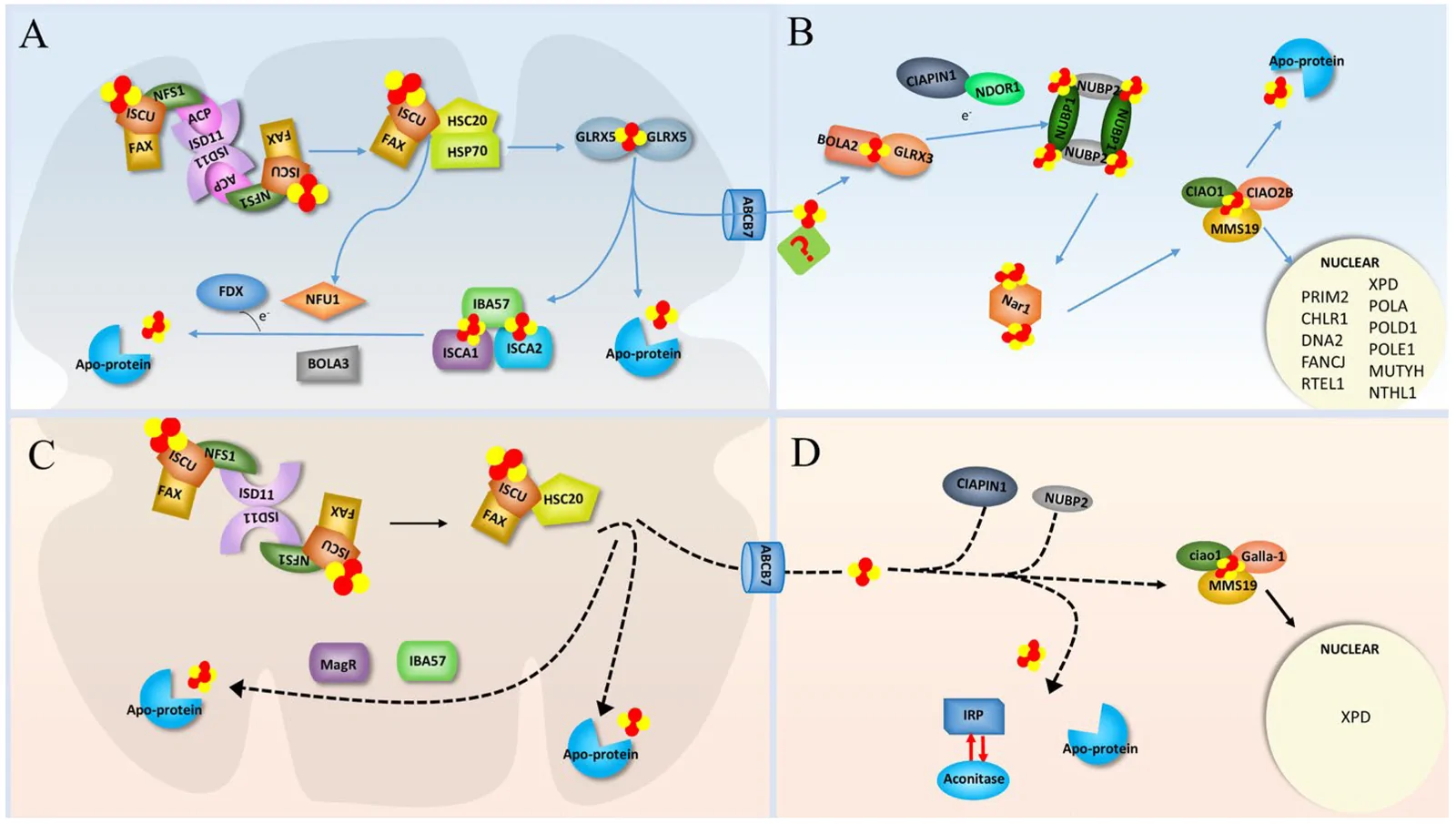
Core protein network for the biosynthesis and transport of Fe-S clusters. This figure systematically illustrates the key proteins and their cooperative relationships involved in the entire process, from the assembly of Fe-S clusters within mitochondria to their final delivery to various apoproteins. It outlines the core components of the mitochondrial and cytosolic Fe-S cluster synthesis pathways (ISC and CIA, respectively) in both mammalian (**A**,**B**) and insect (**C**,**D**) systems. Solid arrows indicate experimentally confirmed relationships, while dashed arrows indicate steps that have not yet been experimentally verified. ISCU: Iron-Sulfur Cluster Scaffold Protein; NFS1: Cysteine Desulfurase; ISD11: Iron-sulfur Protein Biogenesis Desulfurase-Interacting Protein 11; ACP1: Acyl carrier protein 1; FAX: Frataxin; FDX: Ferredoxin; FDXR: Ferredoxin Reductase; Hsp70: Heat Shock Protein 70; Hsc20: Heat Shock Cognate Protein 20; GLRX5: Glutaredoxin 5; ABCB7: ATP-binding Cassette Sub-family B Member 7; ISC system: Iron-Sulfur Cluster Assembly system; CIA system: Cytosolic Iron-Sulfur Protein Assembly pathway; ISCA1: Iron-Sulfur Cluster Assembly 1; ISCA2: Iron-Sulfur Cluster Assembly 2; IBA57: Iron-Sulfur Cluster Assembly Factor IBA57; NFU1: Nitrogen Fixation Subunit U-like protein 1; BOLA3: BolA Family Member 3; BOLA2: BolA Family Member 2; GLRX3: Glutaredoxin 3; NUBP1: Nucleotide-Binding Protein 1; NUBP2: Nucleotide-Binding Protein 2; NDOR1: NADPH-dependent Diflavin Oxidoreductase 1; CIAPIN1: Cytokine-Induced Apoptosis Inhibitor 1; Nar1: Nuclear Architecture Related 1; CIAO1: Cytosolic Iron-sulfur Protein Assembly Protein 1; CIAO2B: Cytosolic Iron-sulfur Protein Assembly Protein 2B; MMS19: Methyl Methanesulfonate Sensitivity 19; PRIM2: DNA primase subunit 2; CHLR1: CHL1-related protein 1; DNA2, DNA replication helicase/nuclease 2; FANCJ: Fanconi anemia group J protein; RTEL1, regulator of telomere elongation helicase 1; XPD, general transcription and DNA repair factor IIH helicase subunit XPD; POLA: DNA polymerase α catalytic subunit; POLD1, DNA polymerase δ catalytic subunit; POLE1: DNA polymerase ε catalytic subunit; MUTYH: mutY DNA glycosylase; NTHL1, Endonuclease III-like protein 1 (DNA glycosylase).

The [2Fe-2S] cluster serves as the fundamental building block for the synthesis of various Fe-S clusters. In eukaryotes, its biosynthesis occurs on the scaffold protein Iron-Sulfur Cluster Scaffold Protein (ISCU) within the mitochondrial matrix [31]. In this process, sulfur is supplied by the cysteine desulfurase complex, which is composed of Cysteine Desulfurase (NFS1), Iron-sulfur Protein Biogenesis Desulfurase-Interacting Protein 11 (ISD11), and Acyl carrier protein 1 (ACP1) [32], while the iron source is supplied by the iron chaperone protein Frataxin (FAX) [33] Assembly is completed through an electron transfer process mediated by Ferredoxin (FDX), which is reduced by Ferredoxin Reductase (FDXR) [34,35]. Subsequently, the assembled [2Fe-2S] cluster is released from ISCU via the mediation of the Heat Shock Protein 70-Heat Shock Cognate Protein 20 (Hsp70-Hsc20) chaperone system [36]. The released cluster can then be received by Glutaredoxin 5 (GLRX5) for subsequent transformations within the mitochondrion. Alternatively, it can be exported to the cytosol in a glutathione-bound form via the transporter ATP-binding Cassette Sub-family B Member 7 (ABCB7), where it serves as a precursor for the cytosolic Fe-S cluster assembly pathway [37,38]. The [4Fe-4S] cluster biogenesis proceeds via distinct mitochondrial (ISC system) (Figure 2A) and cytosolic pathways (CIA system) (Figure 2B). The ISC system is orchestrated by the Iron-Sulfur Cluster Assembly 1-Iron-Sulfur Cluster Assembly 2 (ISCA1-ISCA2) heterodimer, which, with electrons supplied by Iron-Sulfur Cluster Assembly Factor IBA57 and FDX, reconstitutes two [2Fe-2S] clusters into one [4Fe-4S] cluster [39,40]. This nascent cluster is subsequently delivered by carrier proteins such as Nitrogen Fixation Subunit U-like protein 1 (NFU1) to mitochondrial target apoproteins. BolA Family Member 3 (BOLA3) likely functions in this process to maintain the cluster in a reduced state [41,42]. In the cytosolic pathway, the [2Fe-2S] cluster exported by ABCB7 is stabilized and transferred by the BolA Family Member 2- Glutaredoxin 3 (BOLA2-GlRX3) complex to the scaffold formed by Nucleotide-Binding Protein 1- Nucleotide-Binding Protein 2 (NUBP1- NUBP2) [43,44]. Utilizing reducing equivalents provided by the NADPH-dependent Diflavin Oxidoreductase 1- Cytokine-Induced Apoptosis Inhibitor 1 (NDOR1-CIAPIN1) electron transfer chain, the [2Fe-2S] clusters are assembled into a [4Fe-4S] cluster [45,46]. This mature cluster is ultimately delivered by Nuclear Architecture Related 1 (Nar1) to the CIA-targeting complex, which is composed of Cytosolic Iron-sulfur Protein Assembly Protein 1 (CIAO1), Cytosolic Iron-sulfur Protein Assembly Protein 2B (CIAO2B), and Methyl Methanesulfonate Sensitivity 19 (MMS19), ensuring its precise insertion into cytosolic and nuclear apotarget proteins [47].

The Fe-S cluster biosynthetic pathway in insects exhibits distinct specificity compared to other eukaryotes. The results of the studies indicate that the NFS1, ISD11, and ISCU of insects form a stable complex [48]. Frataxin has been shown to facilitate the delivery of Fe^2+^ to the ISCU, thereby promoting the assembly of Fe-S clusters. It is noteworthy that, in comparison with other eukaryotes, the FAX in *Drosophila* exhibits significant evolutionary adaptations in both structure and function. The results demonstrate that the FAX from *Drosophila* exhibits enhanced structural stability and stronger ferrous ion (Fe^2+^) binding affinity, suggesting a potentially higher efficiency as an iron chaperone. The results demonstrate that the *Drosophila* ISCU homolog exhibits superior solubility and anti-aggregation properties in vitro, maintaining stable functional dimeric conformations even at elevated Fe^2+^ concentrations [49,50]. Furthermore, in *Aedes albopictus*, the core Fe-S cluster biosynthetic proteins NFS1 and FAX show high homology with their mammalian homologs; however, the sequence similarity of the key chaperone proteins ISCU and Hsc20, responsible for “assembly” and “transfer,” is below 25% [51]. This feature suggests that the Fe-S cluster synthesis and delivery pathways in insects may possess greater stability, which could represent an adaptive mechanism of insects as ectotherms. Fluctuations in environmental temperature directly and significantly affect insect metabolic rates and mitochondrial function, resulting in a U-shaped dynamic change in reactive oxygen species (ROS) production: ROS levels are lowest within the optimal temperature range (12–18 °C), while they surge under both low- and high-temperature extremes [52]. Fe-S clusters are highly sensitive to oxidative damage [53]. Consequently, the periodic oxidative stress induced by temperature fluctuations imposes an increased demand for de novo Fe-S cluster synthesis in insects. However, research on the biogenesis mechanism of insect Fe-S clusters is significantly lagging behind that of mammalian systems. This process involves numerous proteins, including ACP, HSP70 (Figure 2C), and the majority of components of the cytoplasmic Fe-S cluster CIA system (Figure 2D). However, the systematic identification of these proteins remains to be fully elucidated.

## 4. Functional Roles of Iron-Sulfur Clusters Within Proteins in Insects

### 4.1. Energy Hub: Fe-S Clusters in the Electron Transport Chain and TCA Cycle

**Figure 3:**
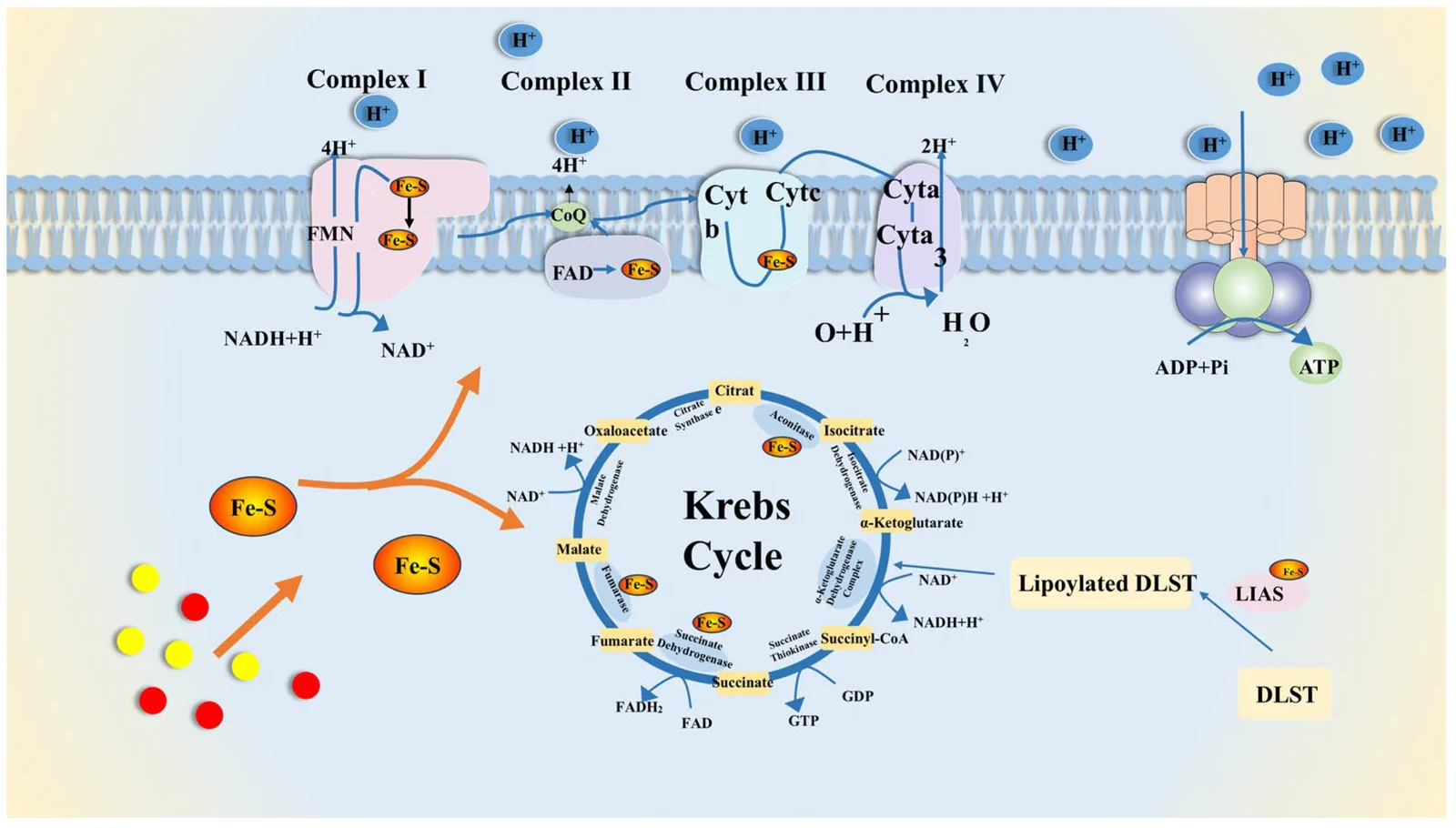
The following schematic diagram illustrates the relationship between Fe-S clusters and energy metabolism. Fe-S clusters have been demonstrated to participate in the mitochondrial electron transport respiratory chain and the tricarboxylic acid (TCA) cycle via multiple key pathways, thereby exerting a significant influence on cellular energy (ATP) production. The orange arrows indicate the two major fates of Fe-S clusters in energy production: the electron transport chain (ETC) and the tricarboxylic acid (TCA) cycle. DLST: dihydrolipoamide S-succinyltransferase. LIAS: lipoic acid synthetase.

Fe-S clusters are present at multiple critical catalytic sites within Complexes I–III of the electron transport chain (ETC) and the tricarboxylic acid (TCA) cycle, thereby positioning them at the intersection of the two principal energy-generating routes. This distinctive location makes them a potential target for intervention in energy metabolism (Figure 3).

The targeting of Fe-S cluster biosynthesis has been demonstrated to disrupt multiple Fe-S cluster-dependent metabolic nodes, thereby inducing the collapse of energy metabolism. This provides a robust theoretical foundation for the design of novel green insecticides based on the regulation of energy metabolic networks. In the electron transport chain, Fe-S clusters are widely distributed in key enzymes of the mitochondrial respiratory chain, including Complex I (NADH dehydrogenase), Complex II (succinate dehydrogenase), and Complex III (ubiquinol-cytochrome c reductase) [54]. Disruption of Fe-S cluster biosynthesis has been shown to markedly reduce the expression of NDUFS (NADH dehydrogenase [ubiquinone] iron-sulfur protein), a core subunit of Complex I [55]. This leads to an over-80% reduction in Complex I activity in *Drosophila*, thereby causing metabolic dysregulation. Phenotypically, this is reflected in a reduction in maximum walking distance of up to 91% and a sixfold decline in crawling speed [56]. The stability of Fe-S clusters is a critical factor in determining the activity of Complex II. In *Drosophila*, the assembly factor SDHAF3 protects these clusters from oxidative damage; *SDHAF3* knockout disrupts the clusters, leading to SDHB degradation and a reduction in complex II activity by approximately 50% [57]. In contrast to mammalian orthologs, the 5′ UTR of insect *SDHB* mRNA, encoding the Complex II subunit SDHB, harbors a functional iron-responsive element (IRE). It has been established that Fe-S cluster insufficiency elicits a twofold regulatory response on Complex II, involving both structural disintegration and IRP-dependent translational inhibition. This may represent an insect-specific mechanism for coping with perturbations in iron homeostasis [58,59]. The Rieske subunit of Complex III has been found to contain a key [2Fe-2S] cluster [60]. It has been demonstrated that impaired assembly of the Fe-S cluster directly results in rapid turnover of this subunit. Accordingly, in *Spodoptera exigua*, suppression of Rieske subunit expression has been demonstrated to compromise Complex III structural integrity and to lead to a 36.6% decrease in larval ATP levels [61].

In addition to the electron transport chain, it has been established that several of the key enzymes in the TCA cycle are Fe-S cluster-dependent. In *Drosophila*, interference with Hsc20, a molecular chaperone involved in iron-sulfur cluster assembly, has been shown to result in a reduction in aconitase activity of approximately 50% and a significant downregulation of succinate dehydrogenase activity, thereby impairing the TCA cycle [62]. Furthermore, fumarase is [4Fe-4S]-cluster-dependent. The knockout of the iron-sulfur assembly factors Isa1/2 in *Trypanosoma brucei* has been demonstrated to reduce fumarase activity to only 10–25% of its original level [63], a mechanism that may also be conserved in insects.

### 4.2. Reproductive Switch: Fe-S Clusters in the AMPK/TOR Axis and Cell Cycle Control

AMPK and TOR have been shown to constitute a classic antagonistic regulatory axis, wherein AMPK directly suppresses TOR signalling through phosphorylation of RAPTOR, a key subunit of the TOR pathway [64,65]. The involvement of this axis in insect reproduction has been experimentally confirmed across multiple species: AMPK activation in *Anopheles stephensi* has been shown to reduce egg production by 26–40% [66]. TOR pathway inhibition has been demonstrated to significantly decrease per-female fecundity in the tobacco whitefly [67], and in the brown planthopper, AMPK activation has been observed to downregulate both key TOR pathway genes and vitellogenin expression [68]. Fe-S proteins have been demonstrated to be closely linked to the AMPK/TOR signalling axis. It has been demonstrated by previous studies that dihydroartemisinin (DHA) degrades multiple Fe-S cluster proteins, leading to an approximately 2.5-fold increase in p-AMPK levels and a reduction of p-mTOR to about 40% of control values. Conversely, the overexpression of *ISCU*, which encodes a core component of the Fe-S cluster assembly machinery, reverses this DHA-induced AMPK phosphorylation [69]. In the brown planthopper, the knockdown of *ISCA1*, a key Fe-S cluster assembly gene, has been shown to result in a 65.34% decrease in total egg production and an 88.07% reduction in egg hatching rate [70]. However, the mechanistic basis of these effects remains unclear. Taken together, these observations suggest that the functional status of Fe-S proteins, which is governed by Fe-S cluster assembly, may regulate vitellogenin synthesis and deposition by modulating the activity of the AMPK/TOR axis, thereby ultimately determining insect reproductive efficiency.

At the cellular level, the maturation-promoting factor (MPF) complex, composed of Cyclin B and cyclin-dependent kinase 1 (CDK1), plays a critical role in mitotic progression [71]. Disruption of Fe-S cluster assembly proteins in *Drosophila* has been shown to result in a significant decrease in the abundance of Cyclin B protein, with the mitotic index of follicle cells dropping to approximately 1% and a decrease in cell number of approximately 75% [72]. Furthermore, defects in Fe-S cluster assembly proteins in the embryos of *Drosophila* also impair the phosphorylation-dependent activation of CDK1, resulting in aberrant chromosome segregation, spindle defects, and a severely reduced hatching rate [73]. The findings suggest that Fe-S clusters sustain MPF function by maintaining the activity of Fe–S proteins, which act through two parallel pathways: one ensuring stable expression of Cyclin B, and the other promoting phosphorylation-dependent activation of CDK1. However, the mechanism by which Fe-S clusters coordinate these two pathways remains elusive. In order to address this question, the following possible mechanism is proposed. The XPD protein contains both a [4Fe–4S] cluster domain and an ARCH domain that is capable of binding the CDK-activating kinase (CAK) [74]. This suggests that XPD may serve as a key node integrating Fe-S cluster signals with CDK1 activation. The cytosolic iron–sulfur protein assembly (CIA) machinery delivers the [4Fe–4S] cluster to XPD [75,76]. It is hypothesised that this delivery process may sterically hinder the interaction between CAK and XPD, thereby releasing CAK to phosphorylate CDK1. Consequently, the status of Fe-S clusters could be coupled to the MPF function.

### 4.3. Ecdysis and Detoxification Alliance: Fe-S Clusters in the Cytochrome P450 Systems

**Figure 4:**
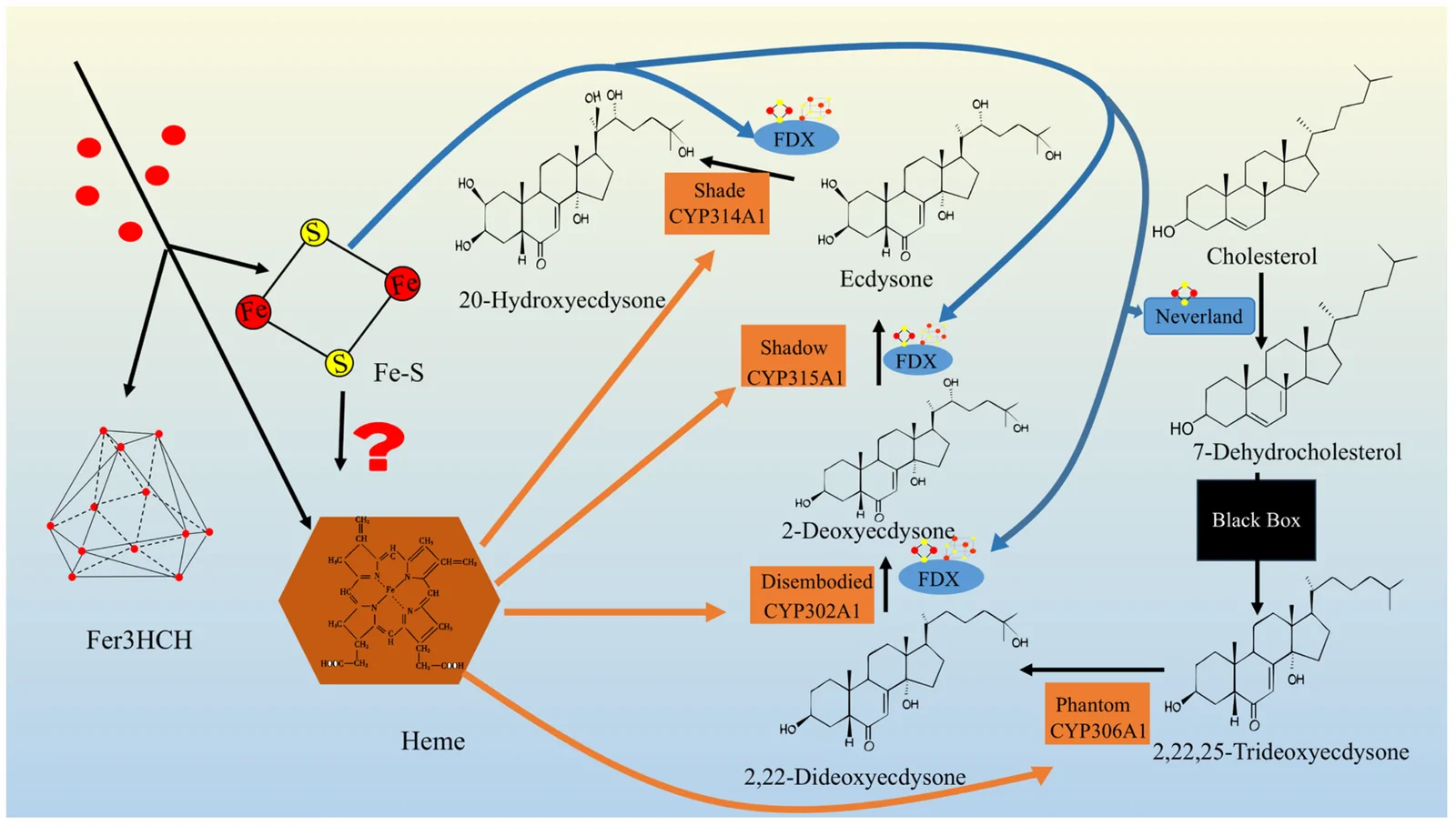
Schematic diagram illustrating the mechanism by which Fe-S proteins regulate the molting process in insects. Iron ions entering the mitochondria follow three metabolic fates: storage by mitochondrial ferritin, utilization for heme synthesis, or assembly into Fe-S clusters. Both heme (orange arrows) and Fe-S clusters (blue arrows) influence molting through multiple downstream pathways. The diagram also highlights an unresolved scientific question: whether the regulatory mechanism of Fe-S clusters on heme synthesis, known in mammals, is conserved in insects.

Recent studies have revealed that Fe-S clusters, as key metabolic cofactors, affect the cytochrome P450 enzymes (P450s) through a dual direct–indirect pathway, thereby participating in two core functions in insects: molting and detoxification adaptation. In the direct pathway, Fe-S clusters serve as the active center of ferredoxin (FDX), providing electron support to activate mitochondrial P450s. For example, in *Helicoverpa armigera*, the Fe-S cluster-binding region of the FDX protein coordinates with a [2Fe-2S] cluster via specific amino acid residues to form a stable electron-transfer module, and through its spatial conformation, it specifically recognizes the redox partner domains of mitochondrial P450s (CYP302A1, CYP315A1, and CYP314A1), ensuring precise electron transfer [77]. In the indirect pathway, Fe-S clusters may influence P450 activity by regulating heme synthesis via the IRP1-ALAS axis [78,79]. Disruption of the gene encoding ALAS, the rate-limiting enzyme of heme synthesis, in *Drosophila* results in ecdysone levels falling below the detection limit (2.5 pg/larva) and a pupation rate of 0% at the third-instar larval stage [80]; similarly, disruption of the key Fe-S cluster assembly gene *frataxin* also leads to a marked reduction in 20E levels and developmental arrest [81], suggesting that the two processes may share a common regulatory pathway. Furthermore, the Neverland protein, which participates in 20E synthesis, itself contains an Fe-S cluster [82]. Collectively, these lines of evidence support a critical role for Fe-S proteins in hormone synthesis (Figure 4).

Notably, the functional organization of insect FDX contrasts sharply with that in mammals. Mammals possess two highly functionally differentiated FDXs, each dedicated exclusively to either Fe-S cluster biosynthesis or steroid hormone synthesis [83]. In contrast, although insects also have two FDX homologs, they exhibit a unique pattern of partial functional overlap. In *Drosophila*, disruption of either *FDX* gene leads to developmental delay, with 20E levels reduced by 87% and 83%, respectively, indicating that both are directly involved in ecdysone synthesis; however, disruption of either gene alone does not severely impair the overall activity of Fe-S cluster-containing proteins [81], suggesting the existence of a compensatory redundancy in the fundamental function of Fe-S cluster synthesis. This evolutionary strategy is distinctly different from the fine-tuned division of labor seen in mammals and may reflect an adaptive choice by insects to meet the specific demands of rapid metamorphosis for hormonal fluctuations while maintaining metabolic robustness.

Cytochrome P450 enzymes that participate in insect molting are also central to the detoxification network [84,85,86,87]. We therefore further hypothesize that Fe-S clusters involved in insect detoxification through their effects on P450s. Preliminary evidence shows that knockdown of *ISCA1*, a gene involved in Fe-S cluster synthesis, in *Nilaparvata lugens* significantly downregulates the expression of the detoxification-related P450 genes *CYP6CS1v2* and *CYP439A2* [70,88,89]. However, direct functional validation remains to be performed.

In summary, Fe-S proteins, with Fe-S clusters as their essential cofactors, integrate the regulation of P450s via both electron transfer and heme synthesis through the dual pathway. The insect-specific functional redundancy of FDX not only deepens our understanding of the regulatory mechanisms underlying ecdysone synthesis but also provides a theoretical entry point for developing novel insecticides that target this pathway to simultaneously interfere with metamorphosis and reduce detoxification capacity.

### 4.4. Photomagnetic Navigation: Fe-S Clusters Couple Magnetoreception and Circadian Rhythms

**Figure 5:**
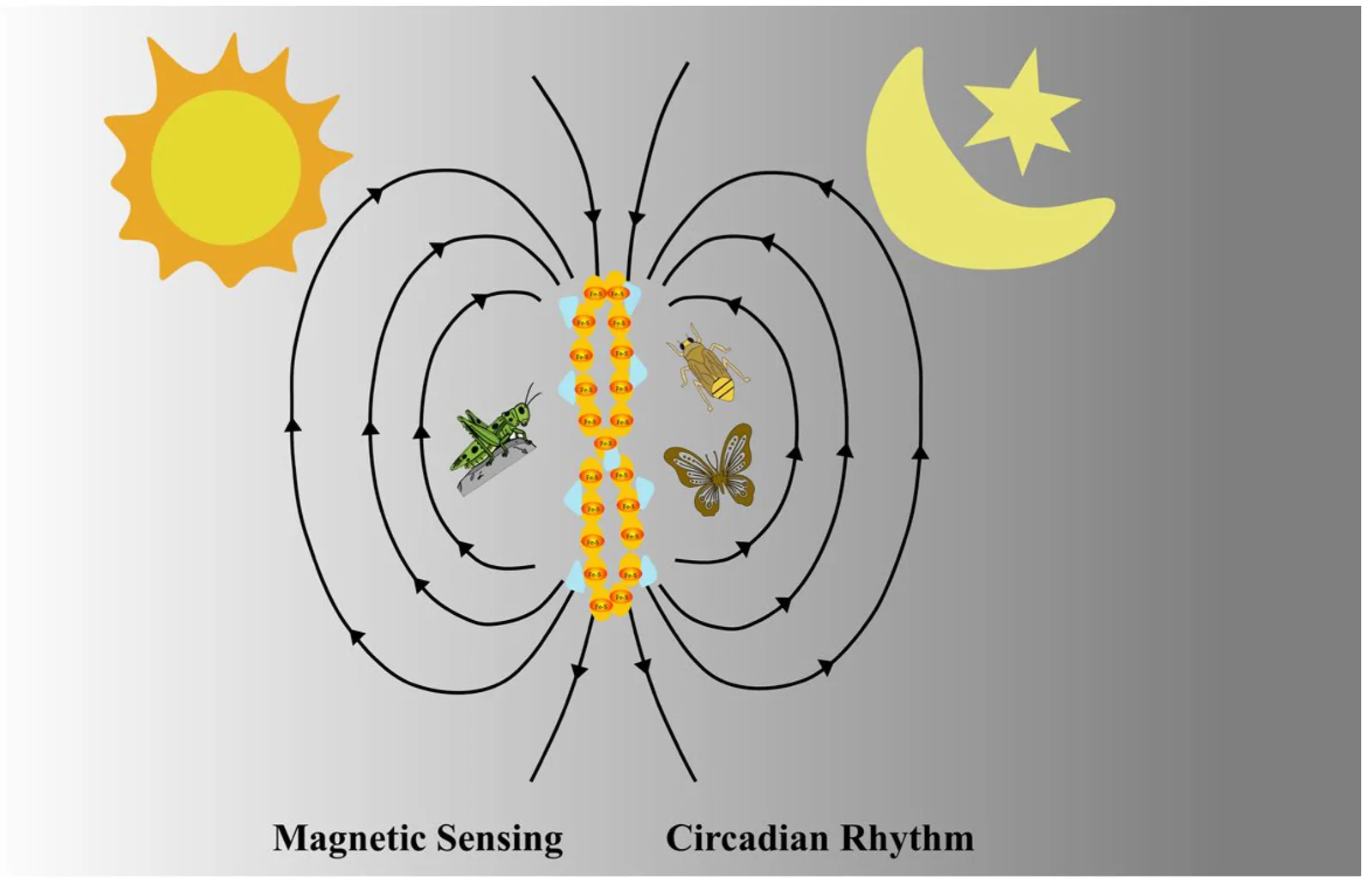
Schematic diagram of Fe-S clusters involved in the photomagnetic coupling mechanism. The Magnetoreceptor (MagR) forms a Fe-S cluster-containing complex with Cryptochrome (Cry), which closely links magnetic sensing with circadian rhythm, thereby providing a precise spatiotemporal coordination basis for complex behaviors of insects such as migration and navigation.

Fe-S clusters have been shown to play pivotal roles in both magnetic sensing and circadian rhythm in insects (Figure 5). In magnetic sensing, the Fe-S cluster-binding protein ISCA1 (MagR) coordinates Fe-S clusters through conserved cysteine residues and assembles with the photoreceptor cryptochrome (Cry) into an intrinsically magnetic multimeric complex [90,91]. This complex directly mediates insect orientation to the geomagnetic field. Disruption of *frataxin*, a gene essential for Fe-S cluster assembly, significantly impairs the geomagnetic orientation behavior of the *Nilaparvata lugen* [92], providing the first evidence that intact Fe-S cluster biogenesis is required for insect geomagnetic sensing. Meanwhile, *MagR* expression oscillates in a circadian manner synchronized with *Cry* [93], whereas dysfunction of key Fe-S cluster biosynthesis genes (*IscS*, *IscU*, *IscA1*, *Iba57*, and *Nubp2*) perturbs the rhythmic fluctuations of core clock proteins, leading to circadian disruption [94]. The Fe-S cluster-binding region of MagR has been rigorously demonstrated to be essential for the formation of a stable MagR–Cry complex [95]. Given its involvement in both processes, we propose that Fe-S clusters may serve as a molecular hub that integrates magnetic sensing and circadian rhythm functions. Within this complex, light signals are transduced via the FAD cofactor of Cry [96], whereas the perception and transduction of magnetic signals depend on the Fe-S clusters bound to MagR. This integrative mechanism may allow insects to coordinate temporal information from the photoperiod with spatial cues from the geomagnetic field within the circadian framework, thereby providing a precise spatiotemporal basis for complex behaviors such as migration and navigation.

## 5. Conclusions

This review systematically integrates existing evidence and proposes that Fe-S clusters and their associated proteins in insects do not participate independently in a single physiological process but rather support a series of critical life activities in a developmentally ordered manner. During the larval stage, the functions of Fe-S clusters are exerted through Fe-S proteins, which perform two roles. First, as core components of respiratory chain complexes I, II, and III and of aconitase in the tricarboxylic acid cycle, they ensure efficient ATP synthesis, providing an energy foundation for all subsequent physiological activities [97]. Second, via the P450 enzyme system, they participate in the detoxification of xenobiotics. Multiple lines of evidence indicate that detoxification enzyme expression levels are highest during the larval stage, consistent with the heightened susceptibility of larvae to external stress [98,99,100]. The energy production and detoxification capacity at this stage jointly lay a material foundation for adult survival and reproduction. During the transition from larva to adult, Fe-S clusters provide electrons for the synthesis of ecdysteroids via ferredoxin, driving molting hormone production and thereby regulating this critical developmental transition. After entering the adult stage, the functions of Fe-S proteins further differentiate. On the one hand, they regulate reproductive development by modulating the stability of the AMPK/TOR signaling pathway and cell cycle-related proteins such as CIAPIN1 and XPD. On the other hand, during migration, Fe-S clusters, as the core components of the MagR–Cry complex, integrating photoperiodic information with the direction of the geomagnetic field to achieve precise orientation and environmental perception. Thus, Fe-S proteins exhibit a developmental stage-dependent functional progression—from energy metabolism and detoxification in larvae, to ecdysteroid synthesis during metamorphosis, and to reproduction and magnetoreception in adults. This layout likely represents a survival strategy evolved by insects under the pressures of a short life cycle, high reproductive rate, and environmental selection.

Notably, insects exhibit significantly different characteristics from mammals in the regulatory mechanisms related to Fe-S proteins. Insect *SDHB* displays a unique IRP/IRE regulatory mechanism not found in mammals. Together with the previously described -IRP1(Fe-S cluster)-ALAS-heme regulatory axis, this indicates a tighter coupling among iron metabolism, the respiratory chain, and heme synthesis in insects. Another significant difference lies in the functional differentiation of ferredoxins. In insects, both ferredoxin isoforms participate simultaneously in ecdysteroid synthesis and Fe-S cluster assembly. This is consistent with the short life cycle, rapid growth, and high reproductive rate of insects, thereby establishing a tighter link between ecdysteroid synthesis and Fe-S cluster assembly to support efficient development [101]. In contrast, in mammals, the two ferredoxins have specialized functions [102]. Systematic elucidation of these differences highlights the uniqueness of the regulatory network of Fe-S clusters in insects.

In summary, this review systematically synthesizes existing evidence and highlights the central role of Fe-S clusters in multiple insect physiological processes, including energy homeostasis, reproductive capacity, ecdysis, detoxification metabolism, and magnetoreception. Nevertheless, several key questions remain unanswered. Although most key proteins involved in mitochondrial Fe-S cluster biosynthesis have been identified, our understanding of the cytosolic Fe-S cluster assembly machinery remains incomplete. Moreover, the existence of insect-specific components in this pathway awaits further investigation. Furthermore, we lack a comprehensive understanding of the tissue- and developmental-stage-dependent expression patterns of Fe-S cluster assembly genes, as well as their responses to environmental stressors in insects. Targeting the Fe-S cluster assembly pathway may exert multimodal physiological disruption in insect pests by simultaneously impairing energy metabolism, developmental transitions, detoxification capacity, and environmental sensing. From a translational perspective, exploiting structural differences between insect and mammalian Fe-S cluster assembly components to design species-selective inhibitors could offer novel targets for next-generation precision pest control.

## Data Availability

No new data were created or analyzed in this study. Data sharing is not applicable to this article.
